# Medical syncretism in Yogyakarta: what do the practitioners get?

**DOI:** 10.1186/1471-2458-14-S1-O32

**Published:** 2014-01-29

**Authors:** Retna Siwi Padmawati, Jens Seeberg, Laksono Trisnantoro

**Affiliations:** 1 Faculty of Medicine, Universitas Gadjah Mada, Yogyakarta 55281, Indonesia

## Background

Medical syncretism involves activities conducted by healers who are mixing or blending different treatment strategy and explanatory models, is prevalent in the modern world. In medical pluralism, the pattern demonstrates that biomedicine exerts dominance over alternative medical system. The rigid clinical practice guidelines derived from the current best evidence of systematic reviews and meta-analyses involving many randomised-controlled trials (RCTs), have strictly guided physicians in managing patients’ diseases. In poor neighbourhood of Yogyakarta, it is not only the health seekers who combine biomedicine and traditional medicines, but some biomedical practitioners combined their techniques with alternative or traditional system. This paper aimed to describe why biomedical practitioners practicing medical syncretism; to explore what are their underlying practices; and what are their roles in medical pluralism.

## Materials and methods

The research was part of a bigger project on health system reforms and ethics among private practitioners. It was conducted in poor neighbourhood of Yogyakarta with 30 health resources/institutions and 29 families as informants.

## Results

Some doctors and nurses were combining their treatments with acupunctures, traditional Chinese Medicines (TCM), bekam, prayers, reiki and herbal medicines. The uses were adjusted to the diagnosis and the capacity to pay. The most common reason in blending the systems was to help patients to remedy their illnesses because alternative and traditional medicines were safer in the hand of the biomedical providers. Other reasons were providing one stop treatment, to compete with alternatives, to attract patients, and the limitation in biomedicine. Biomedical providers also shared with the patients on the efforts of finding the best cure for their conditions (cocok). Patients had been attracted to visit the said providers as they have endorsed the use of alternative and traditional medicines; creating and confirming their roles for medical pluralism. There was no policy in place for such practices and little attention was given by the Indonesian Medical Doctors’ Association to the practices.

## Conclusions

Although medical syncretism seems to be practical to certain health providers, it is questionable in the era of universal health coverage in Indonesia.

